# 6-Chloro-1-(3,5-dimethyl­phenyl­sulfon­yl)-1*H*-benzimidazol-2(3*H*)-one

**DOI:** 10.1107/S1600536808042694

**Published:** 2008-12-20

**Authors:** Fiorella Meneghetti, Gabriella Bombieri, Patrizia Logoteta, Laura De Luca

**Affiliations:** a Institute of Pharmaceutical and Toxicological Chemistry, "P. Pratesi", University of Milano, via L. Mangiagalli, 25, 20133-Milano, Italy; b Department of Pharmaceutical Chemistry, University of Messina, viale Annunziata, 98168-Messina, Italy

## Abstract

The title compound, C_15_H_13_ClN_2_O_3_S, is one of a series of *N* ^1^-benzyl-1,3-dihydro-2*H*-benzimidazol-2-one derivatives, a new class of non-nucleoside HIV-1 reverse transcriptase inhibitors. The dihedral angle between the two pharmacophoric groups, the dimethyl­benzene ring and the benzimidazolone ring system, is 88 (1)°, giving a butterfly-like conformation to the mol­ecule. The mol­ecular packing is characterized by a bifurcated N—H⋯(O,O) hydrogen bond and short Cl⋯O contacts of 3.122 (2) Å. In addition, π–π stacking of the benzimidazolone rings is also present, with inter­planar separations of 3.95 (1) Å.

## Related literature

For the role of the substituents on the benzene nucleus in anti-HIV-1 compounds, see: Barreca *et al.* (2007 ▶). For related literature, see: Barreca *et al.* (2005 ▶); Beddoes *et al.* (1986 ▶); Liu *et al.* (2007 ▶).
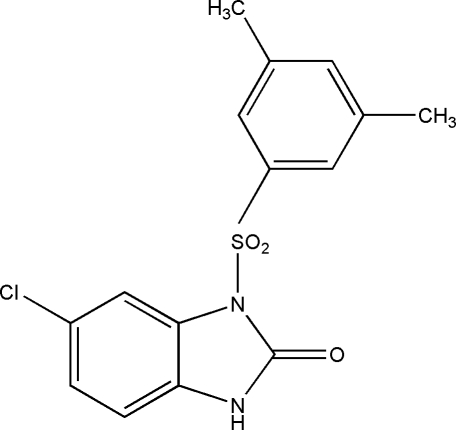


## Experimental

### 

#### Crystal data

C_15_H_13_ClN_2_O_3_S
*M* *_r_* = 336.78
Monoclinic, 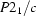

*a* = 12.173 (3) Å
*b* = 14.036 (3) Å
*c* = 8.949 (2) Å
β = 95.77 (2)°
*V* = 1521.3 (6) Å^3^
*Z* = 4
Mo *K*α radiation
μ = 0.40 mm^−1^
*T* = 293 (2) K
0.5 × 0.4 × 0.3 mm

#### Data collection

Enraf–Nonius CAD-4 diffractometer
Absorption correction: none
3964 measured reflections
3634 independent reflections
3214 reflections with *I* > 2σ(*I*)
*R*_int_ = 0.014
3 standard reflections frequency: 120 min intensity decay: 1%

#### Refinement

*R*[*F* ^2^ > 2σ(*F* ^2^)] = 0.057
*wR*(*F* ^2^) = 0.133
*S* = 1.19
3634 reflections
199 parameters
H-atom parameters constrained
Δρ_max_ = 0.23 e Å^−3^
Δρ_min_ = −0.27 e Å^−3^

### 

Data collection: *CAD-4 Software* (Enraf–Nonius, 1989 ▶); cell refinement: *CAD-4 Software*; data reduction: *XCAD4* (Harms & Wocadlo, 1995 ▶); program(s) used to solve structure: *SIR92* (Altomare *et al.*, 1994 ▶); program(s) used to refine structure: *SHELXL97* (Sheldrick, 2008 ▶); molecular graphics: *ORTEP-3 for Windows* (Farrugia, 1997 ▶); software used to prepare material for publication: *WinGX* (Farrugia, 1999 ▶).

## Supplementary Material

Crystal structure: contains datablocks I, global. DOI: 10.1107/S1600536808042694/fj2178sup1.cif

Structure factors: contains datablocks I. DOI: 10.1107/S1600536808042694/fj2178Isup2.hkl

Additional supplementary materials:  crystallographic information; 3D view; checkCIF report

## Figures and Tables

**Table 1:** Hydrogen-bond geometry (Å, °)

*D*—H⋯*A*	*D*—H	H⋯*A*	*D*⋯*A*	*D*—H⋯*A*
N2—H2⋯O1^i^	0.86	2.18	2.852 (3)	135
N2—H2⋯O2^i^	0.86	2.39	3.075 (3)	138

Symmetry code: (i) 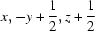.

**Fig. 1.**
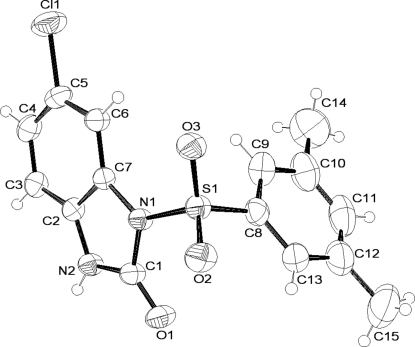
: The molecular structure of the title compound, showing the atom-labelling scheme. Displacement ellipsoids are drawn at the 40% probability level.

**Fig. 2.**
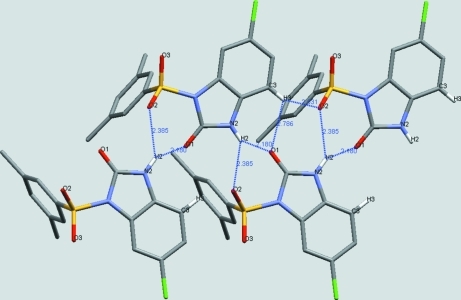
: Packing diagram of the title compound, showing the intermolecular interactions as dotted lines.

## supplementary crystallographic information

### Comment

In the course of previous studies on new anti-HIV agents, some of us reported the synthesis and anti-HIV activity of a series of N1-benzyl-1,3-dihydro-2*H*-benzimidazol-2-ones, a new class of non-nucleoside HIV-1 reverse transcriptase inhibitors (NNRTIs) (Barreca *et al.* 2005). More recently molecular modeling studies led to the discovery of N1-phenylsulfonyl-1,3-dihydro-2*H*-benzimidazol-2-ones as highly potent NNRTIs active at nanomolar concentration (Barreca *et al.* 2007). In this paper we report the results of the X-ray structure determination of 6-chloro-1-(3,5-dimethylphenylsulfonyl)-1,3-dihydro-2*H*-benzimidazol-2-one (I) the most potent derivative of the series, active against wild-type and mutant HIV-1 strains (Barreca *et al.*, 2007). On this respect, its geometrical features defined by X-ray analysis (Fig. 1), could be an useful tool to understand the structure-activity relationship of this class of compounds. The bicyclic part of the molecule consists of an aromatic ring (C2 to C7) and an imidazol-2(3*H*)-one nucleus approximately planar. This bicyclic fragment makes a dihedral angle of 88 (1)° with the dimethylphenyl ring. Such geometry is in agreement with the best docked conformation previously calculated (Barreca *et al.*, 2007) and match well with the pharmacophoric model proposed for the interactions with the macromolecule. As previously observed in other *N*-(phenylsulfonyl)indoles (Liu *et al.*, 2007) and *N*-phenylsulfonamides, (Beddoes *et al.*, 1986) the N atom lone pair eclipses the sulfonyl group; accordingly the corresponding torsion angle O(2)—S(1)—N(1)—C(7) is 48.4 (2)°. In the crystal packing are present two intermolecular hydrogen bonds (Fig. 2) between N2—H2 and O1^I^ at a distance of 2.18 (2) Å, angle 134.2 (2)° and N2—H2 with O2^I^ of 2.39 (2) Å, angle 137.9 (4)°, forming a biforcated linkage with the adjacent molecule at *x*; 1/2 - *y*; *z* + 1/2. The crystal structure is also stabilized by π-π stacking of the benzimidazolone moieties [3.95 (1) Å] and by short intermolecular Cl(1)···O(3)^I^ contacts [3.122 (2) Å].

### Experimental

The compound I has been synthesized as previously reported (Barreca *et al.*, 2007). Single crystals were obtained at room temperature by slow evaporation of a CHCl_3_ solution.

### Refinement

All non-H-atoms were refined anisotropically. Hydrogen atoms were introduced at calculated positions, in their described geometries and allowed to ride on the attached carbon atom with fixed isotropic thermal parameters (1.2Ueq and 1.5Ueq of the parent carbon atom for aromatic H-atoms and methyls H-atoms, respectively).

### Crystal data

**Table:**

C_15_H_13_ClN_2_O_3_S	*F*(000) = 696
*M**_r_* = 336.78	*D*_x_ = 1.470 Mg m^−^^3^
Monoclinic, *P*2_1_/*c*	Mo *K*α radiation, λ = 0.71073 Å
Hall symbol: -P 2ybc	Cell parameters from 25 reflections
*a* = 12.173 (3) Å	θ = 9–10°
*b* = 14.036 (3) Å	µ = 0.40 mm^−^^1^
*c* = 8.949 (2) Å	*T* = 293 K
β = 95.77 (2)°	Prism, colourless
*V* = 1521.3 (6) Å^3^	0.5 × 0.4 × 0.3 mm
*Z* = 4	

### Data collection

**Table:**

Enraf–Nonius CAD-4 diffractometer	*R*_int_ = 0.014
Radiation source: fine-focus sealed tube	θ_max_ = 28.0°, θ_min_ = 3.3°
graphite	*h* = −16→15
Non–profiled ω/2θ scans	*k* = 0→18
3964 measured reflections	*l* = 0→11
3634 independent reflections	3 standard reflections every 120 min
3214 reflections with *I* > 2σ(*I*)	intensity decay: 1%

### Refinement

**Table:**

Refinement on *F*^2^	Primary atom site location: structure-invariant direct methods
Least-squares matrix: full	Secondary atom site location: difference Fourier map
*R*[*F*^2^ > 2σ(*F*^2^)] = 0.057	Hydrogen site location: inferred from neighbouring sites
*wR*(*F*^2^) = 0.133	H-atom parameters constrained
*S* = 1.19	*w* = 1/[σ^2^(*F*_o_^2^) + (0.0188*P*)^2^ + 1.7615*P*] where *P* = (*F*_o_^2^ + 2*F*_c_^2^)/3
3634 reflections	(Δ/σ)_max_ = 0.003
199 parameters	Δρ_max_ = 0.23 e Å^−^^3^
0 restraints	Δρ_min_ = −0.27 e Å^−^^3^

### Special details

**Table:**

Geometry. All e.s.d.'s (except the e.s.d. in the dihedral angle between two l.s. planes) are estimated using the full covariance matrix. The cell e.s.d.'s are taken into account individually in the estimation of e.s.d.'s in distances, angles and torsion angles; correlations between e.s.d.'s in cell parameters are only used when they are defined by crystal symmetry. An approximate (isotropic) treatment of cell e.s.d.'s is used for estimating e.s.d.'s involving l.s. planes.
Refinement. Refinement of *F*^2^ against ALL reflections. The weighted *R*-factor *wR* and goodness of fit *S* are based on *F*^2^, conventional *R*-factors *R* are based on *F*, with *F* set to zero for negative *F*^2^. The threshold expression of *F*^2^ > σ(*F*^2^) is used only for calculating *R*-factors(gt) *etc*. and is not relevant to the choice of reflections for refinement. *R*-factors based on *F*^2^ are statistically about twice as large as those based on *F*, and *R*- factors based on ALL data will be even larger.

### Fractional atomic coordinates and isotropic or equivalent isotropic displacement parameters (Å^2^)

**Table:**

	*x*	*y*	*z*	*U*_iso_*/*U*_eq_	
S1	0.78957 (6)	0.45343 (4)	0.18441 (6)	0.04124 (17)	
Cl1	0.89540 (9)	0.73749 (6)	0.67132 (11)	0.0776 (3)	
O3	0.81132 (18)	0.55252 (13)	0.1756 (2)	0.0524 (5)	
O1	0.80048 (19)	0.25911 (13)	0.3298 (2)	0.0545 (5)	
N2	0.85398 (19)	0.32965 (15)	0.5597 (2)	0.0426 (5)	
H2	0.8602	0.2802	0.6166	0.051*	
N1	0.83211 (18)	0.42285 (14)	0.3607 (2)	0.0371 (4)	
O2	0.83888 (19)	0.38810 (15)	0.0896 (2)	0.0567 (5)	
C2	0.8705 (2)	0.42257 (18)	0.6105 (3)	0.0387 (5)	
C8	0.6470 (2)	0.4341 (2)	0.1704 (3)	0.0508 (6)	
C7	0.85543 (19)	0.48366 (17)	0.4866 (2)	0.0351 (5)	
C1	0.8269 (2)	0.32671 (17)	0.4093 (3)	0.0417 (5)	
C4	0.9024 (2)	0.5555 (2)	0.7717 (3)	0.0519 (7)	
H4	0.9177	0.5817	0.8670	0.062*	
C6	0.8637 (2)	0.58080 (18)	0.5011 (3)	0.0425 (5)	
H6	0.8547	0.6215	0.4188	0.051*	
C3	0.8955 (2)	0.4574 (2)	0.7537 (3)	0.0464 (6)	
H3	0.9074	0.4166	0.8356	0.056*	
C5	0.8864 (2)	0.61419 (19)	0.6469 (3)	0.0491 (6)	
C13	0.6006 (3)	0.3614 (3)	0.0822 (4)	0.0734 (10)	
H13	0.6452	0.3204	0.0334	0.088*	
C9	0.5842 (3)	0.4951 (3)	0.2471 (4)	0.0671 (9)	
H9	0.6174	0.5437	0.3060	0.081*	
C11	0.4255 (4)	0.4091 (4)	0.1443 (5)	0.0941 (15)	
H11	0.3495	0.3998	0.1362	0.113*	
C10	0.4705 (3)	0.4829 (4)	0.2352 (5)	0.0858 (12)	
C12	0.4884 (4)	0.3499 (3)	0.0666 (6)	0.0954 (15)	
C15	0.4332 (5)	0.2721 (4)	−0.0324 (8)	0.152 (2)	
H15A	0.3928	0.2305	0.0274	0.229*	
H15B	0.4884	0.2362	−0.0772	0.229*	
H15C	0.3835	0.3005	−0.1099	0.229*	
C14	0.4004 (4)	0.5506 (5)	0.3165 (7)	0.1339 (17)	
H14A	0.4464	0.5843	0.3924	0.201*	
H14B	0.3454	0.5152	0.3626	0.201*	
H14C	0.3650	0.5953	0.2462	0.201*	

### Atomic displacement parameters (Å^2^)

**Table:**

	*U*^11^	*U*^22^	*U*^33^	*U*^12^	*U*^13^	*U*^23^
S1	0.0549 (4)	0.0401 (3)	0.0290 (3)	0.0002 (3)	0.0055 (2)	0.0037 (2)
Cl1	0.1068 (7)	0.0451 (4)	0.0801 (6)	−0.0057 (4)	0.0058 (5)	−0.0229 (4)
O3	0.0702 (13)	0.0436 (10)	0.0431 (10)	−0.0050 (9)	0.0043 (9)	0.0112 (8)
O1	0.0876 (15)	0.0359 (9)	0.0394 (10)	−0.0034 (9)	0.0039 (9)	−0.0019 (8)
N2	0.0592 (13)	0.0362 (10)	0.0325 (10)	0.0004 (9)	0.0044 (9)	0.0061 (8)
N1	0.0522 (12)	0.0326 (9)	0.0266 (9)	0.0025 (8)	0.0046 (8)	0.0029 (7)
O2	0.0803 (14)	0.0593 (12)	0.0319 (9)	0.0065 (11)	0.0119 (9)	−0.0046 (8)
C2	0.0415 (12)	0.0424 (13)	0.0326 (11)	−0.0008 (10)	0.0060 (9)	0.0002 (9)
C8	0.0551 (16)	0.0537 (16)	0.0421 (14)	−0.0006 (13)	−0.0025 (12)	0.0088 (12)
C7	0.0357 (11)	0.0383 (12)	0.0316 (11)	0.0015 (9)	0.0055 (8)	−0.0022 (9)
C1	0.0570 (15)	0.0357 (12)	0.0327 (11)	0.0021 (11)	0.0068 (10)	0.0021 (9)
C4	0.0551 (16)	0.0606 (17)	0.0398 (13)	−0.0041 (13)	0.0039 (11)	−0.0130 (12)
C6	0.0491 (14)	0.0375 (12)	0.0410 (13)	0.0005 (11)	0.0051 (10)	−0.0003 (10)
C3	0.0521 (15)	0.0540 (15)	0.0332 (12)	−0.0012 (12)	0.0040 (10)	0.0017 (11)
C5	0.0524 (15)	0.0400 (13)	0.0557 (16)	−0.0051 (11)	0.0096 (12)	−0.0138 (12)
C13	0.076 (2)	0.0580 (19)	0.080 (2)	−0.0021 (17)	−0.0227 (19)	0.0045 (17)
C9	0.0559 (18)	0.089 (3)	0.0559 (18)	−0.0019 (17)	0.0029 (14)	−0.0021 (17)
C11	0.060 (2)	0.121 (4)	0.096 (3)	−0.021 (2)	−0.016 (2)	0.039 (3)
C10	0.063 (2)	0.123 (4)	0.072 (2)	0.008 (2)	0.0080 (19)	0.015 (2)
C12	0.081 (3)	0.079 (3)	0.117 (4)	−0.011 (2)	−0.033 (3)	0.016 (3)
C15	0.138 (5)	0.115 (4)	0.186	−0.035 (4)	−0.075 (4)	−0.004 (4)
C14	0.080 (3)	0.185	0.140 (5)	0.021 (4)	0.027 (3)	−0.019 (5)

### Geometric parameters (Å, °)

**Table:**

S1—O3	1.419 (2)	C6—C5	1.388 (4)
S1—O2	1.423 (2)	C6—H6	0.9300
S1—N1	1.6667 (19)	C3—H3	0.9300
S1—C8	1.749 (3)	C13—C12	1.368 (6)
Cl1—C5	1.746 (3)	C13—H13	0.9300
O1—C1	1.210 (3)	C9—C10	1.388 (5)
N2—C1	1.354 (3)	C9—H9	0.9300
N2—C2	1.389 (3)	C11—C12	1.367 (7)
N2—H2	0.8600	C11—C10	1.395 (7)
N1—C7	1.419 (3)	C11—H11	0.9300
N1—C1	1.421 (3)	C10—C14	1.512 (7)
C2—C3	1.376 (3)	C12—C15	1.520 (7)
C2—C7	1.399 (3)	C15—H15A	0.9600
C8—C13	1.375 (4)	C15—H15B	0.9600
C8—C9	1.376 (5)	C15—H15C	0.9600
C7—C6	1.372 (3)	C14—H14A	0.9600
C4—C5	1.385 (4)	C14—H14B	0.9600
C4—C3	1.389 (4)	C14—H14C	0.9600
C4—H4	0.9300		
			
O3—S1—O2	120.41 (13)	C4—C3—H3	121.1
O3—S1—N1	105.25 (11)	C4—C5—C6	123.8 (3)
O2—S1—N1	106.81 (11)	C4—C5—Cl1	119.1 (2)
O3—S1—C8	109.74 (14)	C6—C5—Cl1	117.1 (2)
O2—S1—C8	109.40 (14)	C12—C13—C8	119.6 (4)
N1—S1—C8	103.86 (12)	C12—C13—H13	120.2
C1—N2—C2	111.5 (2)	C8—C13—H13	120.2
C1—N2—H2	124.2	C8—C9—C10	119.0 (4)
C2—N2—H2	124.2	C8—C9—H9	120.5
C7—N1—C1	109.87 (18)	C10—C9—H9	120.5
C7—N1—S1	127.94 (16)	C12—C11—C10	122.8 (4)
C1—N1—S1	120.99 (16)	C12—C11—H11	118.6
C3—C2—N2	130.5 (2)	C10—C11—H11	118.6
C3—C2—C7	121.3 (2)	C9—C10—C11	117.8 (4)
N2—C2—C7	108.2 (2)	C9—C10—C14	119.5 (5)
C13—C8—C9	122.0 (3)	C11—C10—C14	122.7 (4)
C13—C8—S1	120.2 (3)	C11—C12—C13	118.7 (4)
C9—C8—S1	117.7 (2)	C11—C12—C15	119.7 (5)
C6—C7—C2	122.1 (2)	C13—C12—C15	121.5 (5)
C6—C7—N1	132.8 (2)	C12—C15—H15A	109.5
C2—C7—N1	105.1 (2)	C12—C15—H15B	109.5
O1—C1—N2	129.2 (2)	H15A—C15—H15B	109.5
O1—C1—N1	125.6 (2)	C12—C15—H15C	109.5
N2—C1—N1	105.1 (2)	H15A—C15—H15C	109.5
C5—C4—C3	119.6 (2)	H15B—C15—H15C	109.5
C5—C4—H4	120.2	C10—C14—H14A	109.5
C3—C4—H4	120.2	C10—C14—H14B	109.5
C7—C6—C5	115.5 (2)	H14A—C14—H14B	109.5
C7—C6—H6	122.3	C10—C14—H14C	109.5
C5—C6—H6	122.3	H14A—C14—H14C	109.5
C2—C3—C4	117.7 (2)	H14B—C14—H14C	109.5
C2—C3—H3	121.1		

### Hydrogen-bond geometry (Å, °)

**Table:**

*D*—H···*A*	*D*—H	H···*A*	*D*···*A*	*D*—H···*A*
N2—H2···O1^i^	0.86	2.18	2.852 (3)	135 (1)
N2—H2···O2^i^	0.86	2.39	3.075 (3)	138 (1)

Symmetry codes: (i) *x*, −*y*+1/2, *z*+1/2.
